# Molecular analysis of cataract families in India: new mutations in the *CRYBB2* and *GJA3* genes and rare polymorphisms

**Published:** 2010-09-10

**Authors:** Sathiyavedu T. Santhiya, Ganesan Senthil Kumar, Pridhvi Sudhakar, Navnit Gupta, Norman Klopp, Thomas Illig, Torben Söker, Marco Groth, Matthias Platzer, Puthiya M. Gopinath, Jochen Graw

**Affiliations:** 1 Dr. ALM Postgraduate Institute of Basic Medical Sciences, Department of Genetics, University of Madras, Taramani, Chennai, India; 2 Regional Institute of Ophthalmology, Government Eye Hospital, Egmore, Chennai, India; 3 JPM Rotary Eye Hospital and Research Institute, Cuttack, Orissa, India; 4 Helmholtz Center Munich - German Research Center for Environmental Health, Institute of Epidemiology, D-85764 Neuherberg, Germany; 5 Helmholtz Center Munich - German Research Center for Environmental Health, Institute of Developmental Genetics, D-85764 Neuherberg, Germany; 6 Leibniz Institute for Age Research - Fritz Lipmann Institute, Genome Analysis Laboratory, D-07745 Jena, Germany; 7 Manipal Life Sciences Centre, Manipal University, Manipal, India

## Abstract

**Purpose:**

The aim of the study was to resolve the genetic etiology in families having inherited cataracts.

**Methods:**

Families afflicted with congenital/childhood cataracts were registered in Chennai and Orissa (India). Blood samples were collected from the probands and available family members. Selected functional candidate genes were amplified by polymerase chain reaction (PCR) and characterized by direct sequencing. Putative mutations were confirmed in healthy controls.

**Results:**

We observed interesting new polymorphisms of ethnic specificity, some of frequent nature, such as a 3-bp deletion in intron 3 of *CRYBB2* (encoding βB2-crystallin) and IVS1+9 c>t variation in *HSF4* (encoding heat-shock factor 4). Some rare single nucleotide polymorphisms (SNPs) co-segregate with the respective phenotype such as IVS3+120c>a of *CRYBB2*, while M44V of *CRYGD* (encoding γD-crystallin), although found in association with blue dot opacity was seen in a few healthy controls too. We identified two new mutations co-segregating along with the respective cataract phenotype within the families that were not seen in healthy controls from India or Germany. These include two missense mutations; one in *GJA3* (encoding gap junction protein α3, which is also referred to as connexin 46); the mutation affects codon 19 (T19M), and the corresponding phenotype is a posterior-polar cataract. The other missense mutation affects *CRYBB2* (W59C; total cataract). Additionally, a cDNA variation (G54A) identified in a zonular cataract affects a highly conserved splice site of *CRYBB2*. This mutation, however, showed reduced penetrance in the family, which might be explained by different molecular consequences in the affected family members: nonsense-mediated decay of the mutated mRNA might have no clinical phenotype in heterozygotes, whereas the translation of the mutated mRNA is predicted to lead to a small hybrid protein (consisting of 16 amino acids of the βB2-crystallin and 18 new amino-acids), which might have a dominant-negative function in the lens.

**Conclusions:**

This report identifies in families with childhood cataract some new alleles, which may be considered as causative for cataracts. Furthermore, we report some geographically restricted rare polymorphic sites, whose significance might be considered in some context as modifiers or alleles in sensitizing ocular lens toward cataractogenesis.

## Introduction

Childhood cataracts are fairly common, and timely clinical intervention is important for a good visual prognosis. Globally, about 20 million children suffer from blindness; over 50% of them indicate a genetic basis, and one third is familial [1]. In India, cataract accounts for ~12% of childhood blindness [2]. When isolated, it exhibits Mendelian inheritance with autosomal dominance as the commonest mode. Molecular analysis revealed ~60 loci to be associated with several phenoypes of childhood cataracts (for recent reviews see [3,4]). Candidate genes include those coding for αA- and αB-crystallins (*CRYAA*; *CRYAB*), for βA1- or βA4-crystallin (*CRYBA1*, *CRYBA4*), for βB1 - βB3-crystallins (*CRYBB1*; *CRYBB2*; *CRYBB3*), for γC-, γD-, or γS-crystallins (*CRYGC*, *CRYGD*, *CRYGS*), for the beaded filament structural proteins (*BFSP1*; *BFSP2*), for connexin 46 (*GJA3*) or connexin 50 (*GJA8*), for the lens membrane intrinsic protein 2 (*LIM2*) and for the major intrinsic protein of lens fibers (*MIP*). Another group of candidate genes codes for transcription factors (heat shock factor 4, *HSF4*; paired-like homeodomain transcription factor 3, *PITX3*; paired-box gene 6, *PAX6*; eyes-absent 1, *EYA1*, and for a homolog to the musculoaponeurotic fibrosarcoma oncogene, *MAF*). Finally, also genes encoding enzymes were tested (glucoseaminyl(N-acetyl)transferase-2, *GCNT2*; chromatin modifying protein-4B, *CHMP4*). Among them, the crystallin-encoding genes are those having the highest probability to be involved in cataract formation; however, some of them are also associated with other diseases besides cataract [5]. Other known causes of congenital, hereditary cataracts are mutations affecting enzymes of sugar metabolism (e.g. galactokinase 1, *GALK1* [6]) or other metabolic disorders like hyperferritinemia (*FTL*) [7]. Clinical as well as genetic heterogeneity of childhood cataracts has been well established. It is suggested that additional genes or environmental factors might act as modifiers [8]. The identified genes and their mutations give us some insight into the process of maintenance of lens transparency and may increase our understanding of the complexity in age related cataracts.

The present study is an attempt to screen five families (CBE21, DJC1, JEE13, JPM1, and SEC18) with autosomal dominant congenital or childhood cataract for possible mutations in selected candidate genes like *CRYAA*; *CRYBB2*; *CRYGC* and *CRYGD*; *GJA3*; *GJA8,PITX3*, and *HSF4*. We report the identification of three novel mutations - one in *CRYBB2* (W59C) in a family (JPM1) with total cataract, and another in *GJA3* (T19M) in a family (SEC18) with posterior polar cataract and the third one with reduced penetrance in *CRYBB2* (G54A) in a proband (DJC1) with zonular cataract. The probable pathogenicity of these mutations as disease causing molecular lesions is discussed in the light of earlier reports.

## Methods

As part of our ongoing study at the Regional Institute of Ophthalmology (RIO), Government Eye Hospital, Chennai, India, several families having visual impairment caused by hereditary cataracts were registered. One familial case (JPM1) was registered from the Rotary Eye Hospital at Cuttack, Orissa, India. The probands and their relatives were examined by senior pediatric ophthalmologists (P.S. and N.G.) with slit-lamp microscope (Zeiss, Oberkochen, Germany). Family history was recorded according to the information given by available family members. The study adopted the tenets of the Declaration of Helsinki, as family members were enlightened about the study, its outcome and their role in regional language before seeking informed consent as per standard norms. The study was also approved by the Institutional Ethical Committee of Dr. ALM - Post Graduate Institute of Basic Medical Sciences, University of Madras, India.

Blood samples (5–10 ml) were collected from available affected and unaffected family members. Genomic DNA was isolated from peripheral blood using salt extraction to precipitate contaminating proteins as described previously [9]. Genomic DNA was amplified by PCR for the exons (and their flanking regions) of the *CRYAA* [10], *CRYBB2* [11], *CRYGC*, *CRYGD* [12], *GJA8*, *GJA3* [13], *HSF4* [14]), and *PITX3* (Table 1) genes. PCR products were checked in 1.5% agarose gels and purified through Nucleospin columns (Macherey and Nagel, Düren, Germany). Sequencing was done commercially (GATC Biotech AG, Konstanz, Germany; or SequiServe, Vaterstetten, Germany) according to standard procedures.

**Table 1:** Primers for amplification of *PITX3* from genomic DNA.

**Primer**	**Sequence**	**Tm (°C)**	**Size (bp)**
PITX3-Ex1-L1	CTGCCATAAAGTGAATGGGCGC	49–60	278
PITX3-Ex1-R1	TCCAAGGTCCAGCAATAGCTCCTC		
PITX3-Ex2-L1	CCATTACCCTGGTCTGTGTCTTCTCTC	56–60	221
PITX3-Ex2-R1	CCCGCACTGGGGATGAAGC		
PITX3-Ex3-L1	TGGGCGCTCTGTGACCTGC	56–60	273
PITX3-Ex3-R1	CGCGGGTGCGAGTCGC		
PITX3-Ex4-L1	CCTTCAGCCGCTGGGACC	63–67	1000
PITX3-Ex4-R1	TCAAGCGCAACTTTGAATCATCAC		

The mutations were confirmed by the presence/absence of the cleavage site for restriction enzymes or by direct sequencing, if no informative restriction site was available. As controls, 30–100 ethnically matched healthy individuals were used. Additionally, we took 96 randomly chosen, healthy individuals of the KORA Survey 4 (Cooperative Health Research in the Region of Augsburg, Germany), which studied a population-based sample of 4,261 subjects aged 25–74 years over the period 1999–2001 [15].

Copy-number variation analysis was done using PCR, the cloning and counting approach as described previously [16]. Briefly, the following primers were used to amplify the region around exon 2 of *CRYBB2* (UCSC Genome Browser [17]: human genome hg18, chr22:23,947,149–23,947,711): 5′-GAC CTC GTT TTT CCC TCC TC-3′ and 5′-GTG GCA AAA CAG GTA AGG GA-3′. Amplicons were cloned and around 90 clones per sample were sequenced. Copy number was determined by calculating the frequency/ratios of alleles at variations rs16979774, rs7291633, G54A, rs56191632, and rs739315. Obtained allele ratios were searched for statistical significant deviations (Fisher’s exact test) from 1:1 which is the typical situation for two copies per diploid genome (no copy number variability). A copy number, e.g., of three, would result in a ratio of 2:1 / 1:2 or frequency of 0.66 / 0.33.

To predict consequences of mutations or polymorphic sites, the Bioinformatics tool of the ExPASy proteomics server was adopted. For secondary structure predictions, we applied the GOR4 method [18]. To predict splice sites the NetGene2 World Wide Web Server from the Center for Biologic Sequence Analysis was employed (Technical University of Denmark [19]; ). Prediction of regulatory RNA motifs was performed using RegRNA, an integrated web server for identifying the homologs of regulatory RNA motifs and elements against an input mRNA sequence.

## Results

### Family CBE21

**Figure 1:**
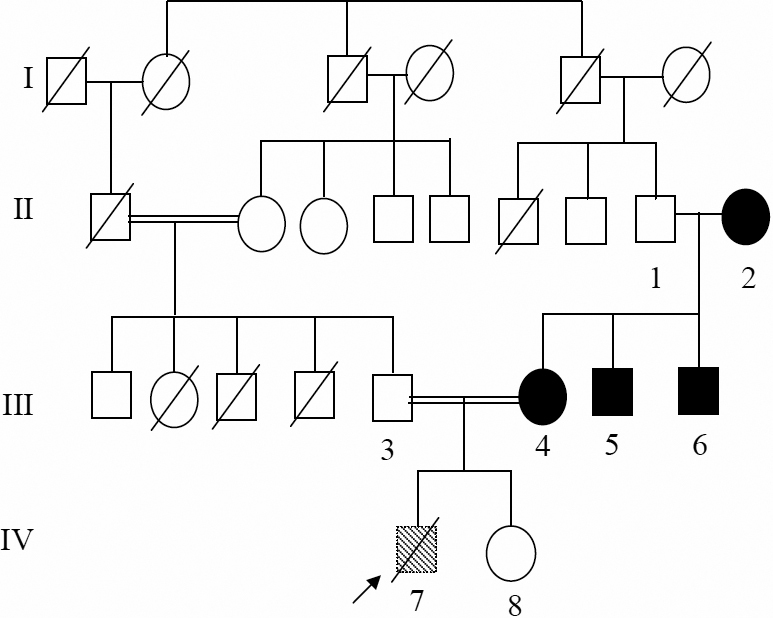
Cataract phenotype in family CBE21. Pedigree of the family CBE21 indicates consanguinity after one generation. The blue dot opacity appeared in the second generation and affects four members of the family. The deceased proband (IV-7) is indicated by an arrow. The numbers below the symbol indicate the laboratory number of the samples.

In family CBE21, the proband was referred from the Institute of Child Health, Chennai, India, as a case of congenital Rubella syndrome to the RIO Egmore Eye Hospital, Chennai. The child was 11 months at the time of case registration, had developmental delay, microcephaly, congenital heart disease, microcornea, nystagmus, and had dense nuclear opacity with peripheral cortical opacity of the lens. The child died at the age of 5 years. No DNA sample was collected from the child. The mother, when examined through slit lamp, was found to have blue dot opacities. One of the mother’s sib (II.6) at the age of 12 years is said to have undergone surgery for cataract. Follow up visit of this family revealed the family to have a second child who is said to be healthy. The pedigree of this family is given in Figure 1, and the case history of the family in Table 2.

**Table 2:** Case history of family CBE21.

**Subject code**	**Relationship**	**Age (Years)**	**VA (L/R)**	**VA (L/R)**	**SLE**
II.2	Proband’s grandmother	55	6/60;6/60	6/6;6/9	Fine blue dot cataract opacity
III.5	Proband’s elder uncle	21	6/60;6/60	6/6;6/9	Fine scattered blue dot opacities
III.6	Proband’s younger uncle	17	6/6;-	-;-	Fine blue dot opacities minimal and scattered

The proband IV.7 was deceased at the age of 5 years. Subject III.6 was operated on at the age of 13 years for removal of a traumatic cataract in his right eye due to a stick injury at the age of 5 years.

### Molecular genetics of Family CBE21

**Figure 2:**
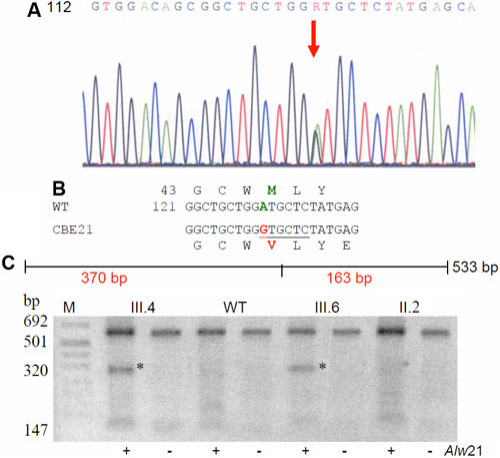
*CRYGD* mutation in the family CBE21. **A**: Sequence analysis of exon 2 of *CRYGD* indicates heterozygosity (arrow) for the mother of the proband (III.4). **B**: Comparison of the wild-type sequence (WT) with the proband’s mother’s sequence (CBE21) demonstrates that the C→T mutation at cDNA position 130 leads to an amino acid exchange from Met to Val at pos. 44 (M44V); moreover, it shows the creation of a new Alw21I restriction site (underlined) in two (III.4 and III.6) of the affected members of the family checked, while the same was absent in the proband’s grand mother (II.2). One of the proband’s uncle (III.5) could not be checked (in the cDNA sequence, the A of the ATG start codon is counted as #1; in the amino-acid sequence, the first Met is counted as #1). **C**: Restriction analysis with (+) or without (-) the enzyme Alw21I in the members of the core family leads to an additional fragment of 370 bp in the mutant DNA (red); it demonstrates the presence of the mutation in the affected mother (III.4) and the affected brother of the proband’s mother (III.6). The asterisks mark the additional band of 370 bp indicating the mutation; the band of 533 bp indicates the undigested DNA. WT represents an independent control from the laboratory; M=marker.

The mother’s genomic DNA was screened for all exons and their flanking sequences of some functional candidate genes (*CRYAA*, *CRYGC*, *CRYGD*, and *PITX3*). This functional candidate gene approach detected a mutation in exon 2 of *CRYGD*. Comparison of the wild-type sequence with that of the proband’s mother demonstrated a heterozygous C→T mutation at position 130 (C130T; Figure 2A), which leads to an amino acid exchange from Met to Val at pos. 44 (M44V; Figure 2B). Further, the mutation creates a new Alw21I restriction site (Figure 2B). Restriction digest showed co-segregation of the genotype with only one other affected member of the family (Figure 2C; III.6). The third affected member was PGM (II.2; 60 years), who also had blue dot opacities, but the restriction site for Alw21l was absent. This restriction site is absent in the proband’s father (III.3) and in unaffected relatives of other cataract probands (n=5; data not shown), it was also not detected in 60 healthy controls from the general population of India but was observed in 6 of the 96 healthy controls (KORA) from Germany. Therefore, it could not be considered as a causative mutation, but may be interpreted as a polymorphism with an allele frequency of 6.2% in the German population.

### Family JEE13

**Figure 3:**
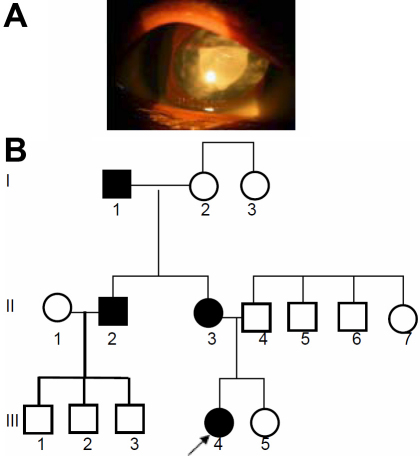
Cataract phenotype in family JEE13. **A**: A lamellar cataract was observed in the 6-year-old proband. **B**: The pedigree of a 3-generation family demonstrates that the cataract appeared in all three generations affecting both sexes; it indicates an autosomal dominant mode of inheritance. The proband is indicated by an arrow.

The proband, a female child of 6 years (as on 2005) of family JEE13 had a triangular central cataract surrounded by cataractous dots and fibers in the lens cortex (Figure 3A). The other eye (LE) was operated earlier on. The proband’s mother (23 years) was examined to have lamellar with peripheral coronary cataract at both eyes and her visual acuity being 3/60. The eyes of the proband’s sib (3 years) however are healthy. One of the mother’s sibs was also affected who has three healthy children (not clinically examined). The pedigree (Figure 3B) demonstrated an autosomal dominant mode of inheritance as the cataract appeared in all three generations affecting both sexes.

### Molecular genetics of Family JEE13

**Figure 4:**
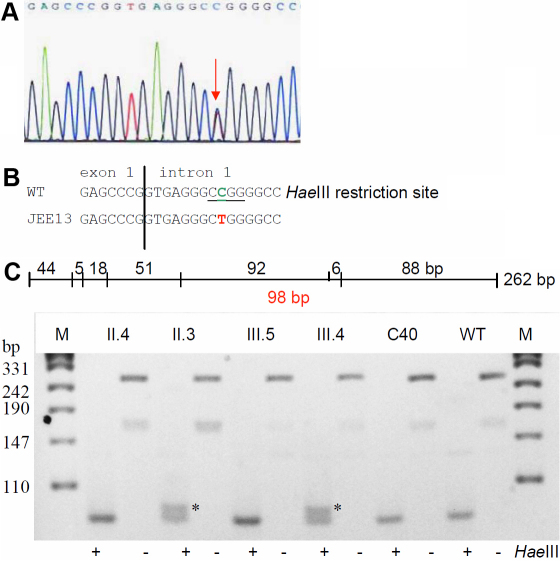
*HSF4* mutation in family JEE13. **A**: Sequence analysis of *HSF4* genomic DNA indicates heterozygosity at position +9 of the first intron (red arrow) of the proband’s *HSF4* gene. **B**: Comparison of the wild-type sequence (AB029347) with the proband’s sequence demonstrates a C→T exchange at position 9 of intron 1. The mutation leads to a loss of a HaeIII restriction site (underlined). **C**: Restriction analysis with (+) or without (-) the enzyme HaeIII in members of the family leads to an additional fragment of 98 bp in the mutant DNA (red); it demonstrates the presence of the mutation in the affected mother (II.3) and in the proband (III.4). The asterisks mark the additional band of 98 bp indicating the mutation; the band of 262 bp indicates the undigested DNA. C40 and WT represent independent controls from our laboratories; M=marker.

Molecular analysis of some functional candidate genes like *CRYAA*, *CRYBB2*, *CRYGC*, *CRYGD*, *GJA3*, *GJA8*, *HSF4*, and *PITX3* revealed a C→T mutation at the beginning of intron 1 of *HSF4* (position #9; Figure 4A); this heterozygous mutation was also present in the affected mother. The mutation leads to a loss of a HaeIII restriction site (Figure 4B) and segregates perfectly in the family (Figure 4C), and was not detected in 96 unrelated controls of the German population. However, among the 35 unrelated and healthy controls from India, 6 of them showed the same restriction pattern as observed in the cataract patients. Therefore, this SNP might be specific for the Indian ethnicity (~17%), and is not associated with cataract formation. This inference is further being supported by the fact that the mutation occurs outside of the conserved recognition sites for splicing; several splice site prediction programs failed to show any influence on splicing.

### Family DJC1

**Figure 5:**
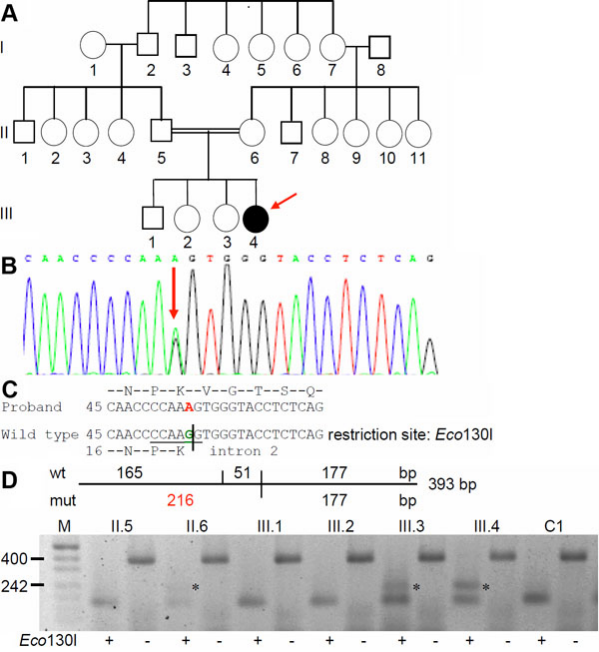
*CRYBB2* mutation in family DJC1. **A**: The pedigree of family DJC1 indicates that a zonular cataract appeared in the youngest daughter of healthy, but consanguineous parents. There is no other report of any type of cataract over three generations. **B**: Sequence analysis of *CRYBB2* genomic DNA indicates heterozygosity for the proband at cDNA pos. 54 (red arrow). **C**: Comparison of the wild-type sequence (Z99916) with the proband’s sequence demonstrates that the G→A exchange at cDNA-position 54 leads to an altered splice site; it is predicted that the mutated mRNA contains at least part of intron 2 (the A of the ATG start codon is counted as #1; in the amino-acid sequence, the first Met is counted as #1). The mutation leads to a loss of an Eco130I restriction site. **D**: Restriction analysis using the enzyme Eco130I creates a larger fragment of 216 bp in the mutant DNA (red); it demonstrates the presence of the mutation in the proband (III.4), but surprisingly also in the healthy mother (II.6) and one healthy sister (III.3) of the proband; the asterisks mark the additional band of 216 bp indicating the mutation. Undigested PCR fragments of 393 bp are indicated by “-” or “+” are digested PCR products. The small fragment of 51 bp in the wild-type DNA is not visible; the two bands of 165 and 177 in the wild type are not separated under the conditions used here. C1 and C2 represent independent controls from the laboratory; M=marker.

The pedigree of the large family DJC1 is shown in Figure 5A. The phenotype “congenital zonular cataract” seems to appear spontaneously in the youngest daughter as proband (1.5 years as on 2005) whose visual acuity (by ivory ball testing) was found to be >6/60 at a distance of 0.5–1.0 m. Both eyes of the proband were operated on, with no other systemic involvement. Her parents were related as first degree cousins. Both the parents and her three sibs underwent complete ophthalmologic check-up and were declared to have regular vision.

### Molecular genetics of Family DJC1

Molecular analysis of functional candidate genes *CRYAA*, *CRYBB2*, *CRYGC*, *CRGYD*, *GJA8*, and *PITX3* revealed a single heterozygous base-pair exchange at the last base of exon 2 of the *CRYBB2* gene (cDNA-G54A; Figure 5B). The mutation does not affect the amino acid in the corresponding codon 18 (it remains Leu; Figure 5C), however, the A instead of the G destroys the common donor splice site, and a splice-site prediction program suggests that the corresponding splice site is completely lost. The most likely next donor splice site might be 338 bp downstream (calculated probability is 79%). If this would be true, the corresponding intronic sequences will be present in the mRNA and translated resulting in 16 novel amino acids; translation will then be terminated by a premature stop codon.

As the mutation leads to a loss of an Eco130I restriction site, its presence or absence was then tested in the core family (father II.5, mother II.6, and the siblings III.1–3) as well as in controls of German origin (n=96) and from unrelated controls of India (n=35). We did not observe the mutation in the German controls, also it was absent in controls from India. Surprisingly, the healthy mother (II.6) and one of the healthy sisters (III.3) showed the same digestion pattern as the proband. Therefore, we sequenced also the corresponding *CRYBB2* exon in the parents (II.5 and II.6) and siblings (III.1–3) of the proband. The mother and the sister III.3 had a small, but significant fluorescence A peak suggestive for copy-number variability of the locus. Therefore, we quantified the allele frequencies of the cDNA-G54A mutation and surrounding SNPs, but the result indicated a 1:1 ratio of the respective alleles typical for a heterozygous single-copy locus. Therefore, this mutation has to be evidently described as having reduced penetrance. Since no operated lens material is available it remains speculative whether the 34-amino acid peptide is actually present in the cataractous lenses or if the mutated mRNA is subjected to nonsense-mediated decay in the clinically healthy family members.

### Family JPM1

**Figure 6:**
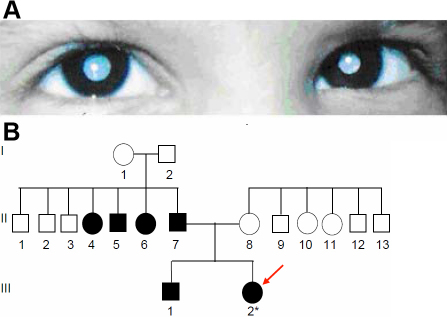
Cataract phenotype in family JPM1. **A**: The eye picture shows a central opacity of both lenses of the female proband (III.2; 2.5 years). **B**: The central lens opacity appeared in 4 out of 7 siblings of seemingly healthy parents (generation I); one of the brothers (generation II) transmitted the cataract to both his offspring (generation III) indicating an autosomal dominant mode of inheritance. The mutation might have originated in the germ cells of the healthy grandparents (generation I).

The proband, a female child of age 2.5 years at the time of registration, was diagnosed to have bilateral congenital cataract (Figure 6A) along with amblyopia and nystagmus in the left eye. There was no other associated ocular or systemic anomaly. She was operated for removal of total cataract in both eyes by lens aspiration, primary posterior capsulotomy and anterior vitrectomy. The mother had no history of any systemic ailments during pregnancy. The child’s developmental milestones were reported to be normal. The child was affected since birth. The father and a male sib were also affected; three of father‘s sibs were said to be affected with cataract. The entire pedigree is shown in Figure 6B.

### Molecular genetics of Family JPM1

**Figure 7:**
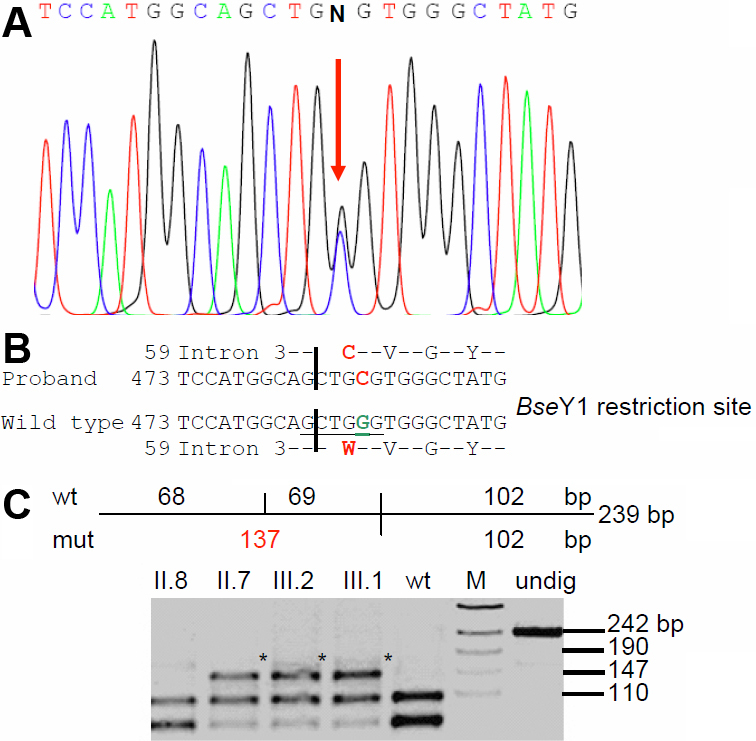
*CRYBB2* mutation in family JPM1. **A**: Sequence analysis of *CRYBB2* genomic DNA indicates heterozygosity in the proband’s father (II.7) at cDNA pos. 177 (red arrow). **B**: Comparison of the wild-type sequence (Z99916) with the proband’s father sequence demonstrates that the G→C exchange at cDNA-position 177 leads to an exchange of Trp by Cys (W59C). The BseY1 restriction site in the wild-type sequence is underlined. **C**: Restriction analysis using the enzyme BseYI leads to an additional fragment of 137 bp in the mutant DNA (red); it demonstrates the presence of the mutation in the affected father (II.7) and the affected brother (III.1) of the proband (III.2). The asterisks mark the additional band of 137 bp indicating the mutation; “undig” is an undigested PCR fragment of 239 bp. WT represents an independent control from the laboratory; M=marker.

Molecular analysis of some functional candidate genes like *CRYAA*, *CRYBB2*, *CRYGC*, *CRYGD*, *GJA8*, and *PITX3* revealed a G→C mutation at the beginning of exon 4 of *CRYBB2* at position 177 leading to an exchange of Trp by Cys (W59C; Figure 7A); the mutation is predicted not to affect the formation of 2nd Greek key motif. Rough computer-assisted prediction of biophysical properties suggests that the hydrophobicity of the area around W59 slightly increases (from 1.4 up to 1.7); although the isoelectric point of the protein is not altered. The mutation destroys a BseY1 restriction site (Figure 7B) which is seen only in affected family members (Figure 7C); it is not present in unrelated controls from India (n=100) or from Germany (n=96). Therefore, this mutation is most likely to be the causative genetic lesion for the cataract in this particular family.

### Family SEC18

**Figure 8:**
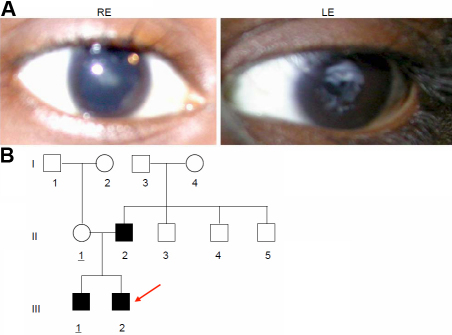
Cataract phenotype in family SEC18. **A**: The posterior polar cataract in the proband (9 years) is demonstrated in the left eye; the right eye is pseudoaphakic. **B**: Pedigree of family SEC18 indicates the occurrence of the cataract in the second generation of two unrelated families; it is fully transmitted to both sons in the third generation.

The proband (III.2) is a male child of 9 years at the time of case registration at RIO in 2007, with a complaint of posterior polar cataract in his left and was pseudoaphakic in his right eye. He underwent cataract surgery at the age of 4y. Presently, his visual acuity is 6/12p with PH 6/12. Subsequently, he had Yag capsulotomy done on his left eye at the age of 9 years (Figure 8A). His elder brother (III.1) is also affected. The pedigree (Figure 8B) demonstrates that the cataract developed spontaneously in the father (II.2) who was operated earlier at the age of 22 years for his right eye. He was 25 years old at the time of his second surgery at left eye for removal of posterior polar cataract. His visual acuity was 6/36 with PH 6/18 at left eye. His pupil is said to be irregular with no other ocular anomalies. Fundus examination revealed his retina with no pathological findings.

### Molecular genetics of family SEC18

**Figure 9:**
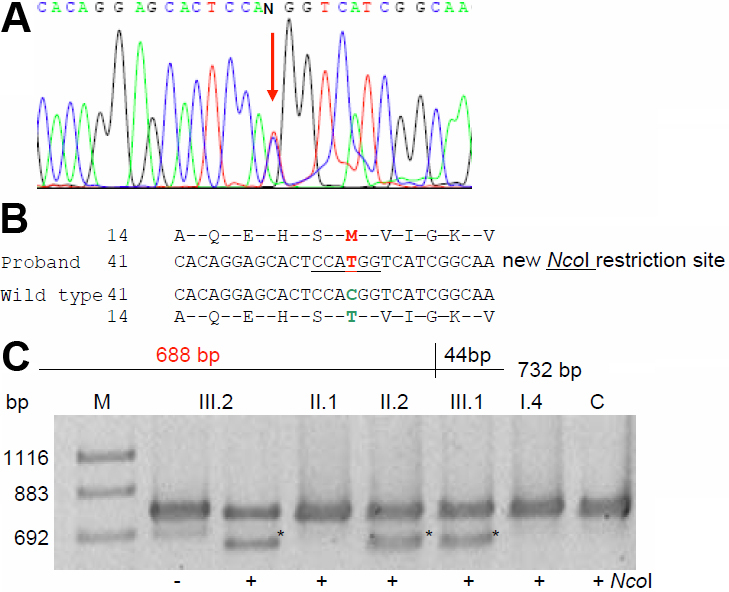
*GJA3* mutation in family SEC18. **A**: Sequence analysis of *GJA3* genomic DNA indicates heterozygosity (red arrow) in the proband. **B**: Comparison of the wild-type sequence with the proband’s sequence demonstrates a C→T exchange at position 56 leading to an amino acid alteration at codon 19 (T19M). **C**: Restriction analysis with (+) or without (-) the enzyme NcoI in members of the family leads to an additional fragment of 688 bp in the mutant DNA (red); it demonstrates the presence of the mutation in the affected father (II.2), in the proband (III.2) and in his brother (III.1). The asterisks mark the additional band of 688 bp indicating the mutation; the band of 732 bp indicates the undigested DNA. WT represents independent control from the laboratory; M=marker.

Molecular analysis of some functional candidate genes (*CRYAA*, *CRYGC*, *CRYGD*, *GJA3*, *GJA8*, and *PITX3*) revealed a C→T mutation of *GJA3* (cDNA position # 56; Figure 9A,B); at the amino-acid level, it exchanges the Thr at codon 19 by a Met (T19M). The mutation forms a new restriction site for NcoI. The mutation segregated within the family (Figure 9C) among the affected members (n=3) and was not observed in unrelated, healthy controls of either Indian (n=100) or German origin (n=96).

The mutation affects the last amino acid of the NH_2_-terminal cytoplasmic domain and is predicted not to alter the first transmembrane domain. However, secondary structure prediction program (GOR4) suggested that the extended-strand and random-coil structure between amino acids 17 and 26 was then changed to an α-helical structure, which runs in the mutant protein from amino acid 5 to 24. It enhances the fraction of the α-helical structure from 42.5% in the wild-type protein to 44.6% in the mutant connexin46.

## Discussion

Here we report a screen of five families with members having congenital or childhood cataracts (CBE21, DJC1, JEE13, JPM1, and SEC18). We identified three mutations being most likely causative for congenital or childhood cataracts in these families from India – one affects the *GJA3* gene encoding connexin 46, the other two affect *CRYBB2* encoding β-crystallin. As the size of the families was rather small we resorted to apply a functional candidate gene approach.

While investigating, two new rare polymorphic sites were documented in some of the candidate genes. The nonsynonymous change M44V in the *CRYGD* gene was identified in two (of four) affected members of a family (CBE21) who suffer from blue dot opacities. This variation was not observed in 60 of the unrelated population controls from India while the same was observed in ~6% of the German controls. In family JEE13, the SNP, IVS1+9 c>t in *HSF4* was observed rather frequently (~17%) in controls of Indian origin. These results suggest ethnic specificity of certain polymorphisms; since these SNPs do not explain the Mendelian type of inheritance, they might act as susceptibility alleles for cataracts in the respective populations. In our screening, several common SNPs were also documented in some of the candidate genes (Table 3) representing coding as well as non-coding regions. SNPs in non-coding regions may influence promoter activity, splicing (in introns), translational efficiency (by different codon usage) or mRNA stability (in 3′-UTR). Therefore, it is evident that SNPs may alter gene function and lead to phenotypic modifications.

**Table 3:** SNPs documented in cataract probands/affected relatives of Indian origin.

**Genes**	**Non coding**	**Family code**	**Coding**	**Family code**
CRYAA	IVS2+27 g>c	CBE21;DJC1	rs60485881	CBE21
CRYBB2	65843t>a;(IVS1+31t>a)	JEE13	54 g>a; K18K	DJC1
	65894a>g;(IVS1+84a>g)	JEE13	rs17842553	SEC18
	71307c>a;IVS3+120c>a)	DJC1; JPM1		
	73644a>g;(IVS3–360a>g)	JEE13;DJC1		
	75738 g>a;(IVS5+9g>a)	JEE13;DJC1	77788 g>a;G161G	JEE13;DJC1
CRYGC	none	none	rs3189020	Sec18
CRYGD	517t>c; IVS2+30t>c	CBE21;DJC1;JPM1;	286t>c; Y16Y	JPM1;DJC1;
		JEE13	365a>g;M43V	CBE21
	570c>t; IVS2+83t>c	CBE21;DJC1;JPM1;	285a>g;R94R	CBE21
		JEE13		JEE13
	553c>t-3′UTR	JEE13		
GJA3	rs968566	SEC18	none	none
GJA8	none	none	none	none
PITX3	none	none	rs17858134	SEC18;CBE21
HSF4	IVS1+9 c>t	JEE13	none	none

In the present study, two new mutations in *CRYBB2* have been identified. In family DJC1; a splice-site prediction program suggests that the G54A mutation of *CRYBB2* disrupts the conserved donor splice site. Unfortunately, this could not be validated further, because experiments on splicing aspects need surgical lens exudates from the proband. This mutation was not observed in the general population of neither Indian (n=35) nor of German (n=96) origin, but it showed reduced penetrance in the family, since two members (the mother and one sister) carry the mutation without clinical findings. Given this ambiguity, it would be interesting to have an animal model for this particular mutation to further understand its mode of action: If the splice site is destroyed, a premature stop codon is present after 16 codons resulting in a short peptide of 34 amino acids. If this peptide is formed, a dominant-negative phenotype might be expected; in case of nonsense-mediated decay of the mutated mRNA, a loss-of-function phenotype without clinical relevance in heterozygotes might be expected. Individual variations among the affected family members might explain the observed reduced penetrance, since the corresponding *Crybb2* knockout mutation in the mouse indicates a less severe phenotype in the homozygotes as compared to the heterozygous dominant point-mutations [20] (the phenotype of heterozygous knockout mutants is not reported and might be considered to be indistinguishable from the wild type).

The second novel mutation, W59C in *CRYBB2*, has been identified to segregate in all affected members of the JPM1 family afflicted with total cataract; no other systemic ailments were obvious. Human βB2-crystallin, has five Trp residues, of which three are in the 2nd Greek key motif at amino acid positions (59, 81, and 84), one in the 4th Greek key motif (151), and the last one in the COOH-terminal extension (194). At position 59, Trp (single letter code W) seems to be a buried residue conserved both in acidic as well as in basic β-crystallins [21] depicting phylogenetic significance. The sequence variation W59C disrupts the conserved Trp residue by substitution of Cys. In human βB2-crystallin, two Cys residues are located at amino acid positions 48 (1st Greek key motif) and 67 (2nd Greek key motif). The putative mutation W59C introduces a third Cys residue in the mutant βB2-crystallin. Theoretically, this might increase the scope for more Cys-mediated crosslinks through di-sulphide bridges which might disrupt the folding and change the topography of the amino acids in the vicinity. As per an earlier report, formation of a disulphide bond involving Cys-37 and Cys-67 of βB2-crystallin has been observed in a human nuclear cataract [22]. Moreover, the microenvironment surrounding the W59 might change in consequence to the mutation, but this hypothesis needs to be verified by appropriate biophysical methods. Interestingly, both the variations W59C (JPM1) and IVS2+1g>a (DJC1) are also in association with an SNP viz., IVS3+120c>a (71307c>a) as was observed earlier in a central nuclear cataract with the mutation W151C of βB2-crystallin [11]. This SNP could therefore act as a molecular tag denoting a marker/modifier for putative mutations in CRYBB2. Putting together the above points, the mutation W59C could well be considered as the molecular lesion for the dominant cataract in the family JPM1.

The appropriate association of β-crystallins into higher-order complexes is critical to the maintenance of lens transparency and a high refractive index [23]. Besides the lens, β-crystallins are expressed in other tissues such as retina, brain, and testis [24,25] implying its role in other biologic functions as well, like elongation of axons during regeneration of retinal ganglion cells [26].

Mutations in human *CRYBB2* have been reported to be associated with variable dominant cataract phenotypes [5,8] like Cerulean cataract [27], Coppock-like cataract [28], Cerulean and sutural cataract [29], a variable phenotype in a Chinese family [30], a dominant congenital cataract in a Chilean family [31], and progressive polymorphic congenital coronary cataract [32]. The mutation Q155X depicted a decrease in protein–protein interaction and decreased ordered structure and stability. However, the partially unfolded protein was found to retain some dimer structure [33]. It is understandable that such heterogeneity in dominant cataracts may be attributed either to ethnic background, or environmental or other modifiers.

Moreover, three other mutations have been identified in *CRYBB2*: W151C in a dominant central nuclear cataract [11] in an Indian family, D128V, affecting exon 5 of the human *CRYBB2* gene in a German family depicting nuclear cataract with an additional ring like cortical opacity [34], and third the mutation S31W co-segregated in a Chinese family with inherited coronary cataract [35].

In mice, three mutant lines have been reported to affect βB2–crystallins, the *Philly* mouse, *AEY2*, and *O377*. In the *Philly* mouse, the progressive opacity involves the nucleus and the anterior sutures [36], while in *Aey2* a progressive opacification of the whole lens sets in by the age of 11 weeks [37], and in *O377* the phenotype is a progressive cataract with a small lens [25]. Further studies of *Crybb2* mutations in animal models might throw light on potential regions of β-crystallin molecule trivial for protein association.

Connexins-46 and −50 are important for proper lens development and differentiation of their fiber cells; they enable contact not only to the anterior epithelial cells, but also among the lens fiber cells [38-42]. Several mutations documented in *GJA3* and *GJA8* substantiate this statement [8]. Here we report a new mutation as T19M in *GJA3*, which was observed in a 9-year-old proband having a posterior polar cataract; the same phenotype and the same mutation (T19M) was confirmed in his elder brother and father, but it was not observed in any of his unaffected family members checked (SEC18). The putative mutation T19M occurs at a highly conserved site both across the family of connexins as well as across species. As per secondary structure prediction program (GOR4), it enhances the fraction of the α-helical structure in the mutant connexin-46.

Altered function and/or expression of specific connexin-encoding genes have been linked to several diseases, including genetic deafness, skin disease, peripheral neuropathies, and cataracts [39]. The cataract-causing mutations in *GJA3* include Asp3Tyr [43], Leu11Ser [44], Val28Met [45], Phe32Leu [46], Arg33Leu [47], Pro59Leu [48], Asn63Ser [49], Arg76Gly [45], Thr87Met [50], Pro187Leu [51], Asn188Thr [52], and c.1137insC causing a frameshift at codon 380 [49]. It is obvious from this list that most mutations identified in human autosomal dominant congenital cataracts (ADCC) so far affect the first half of the protein and the majority of murine cataracts occur in E1 domain [4]. Except the COOH-terminal frame-shift mutation viz., S380fs, all others are missense mutations spread over the NH_2_-terminus (2nos.), M1 (3nos), E1 (3nos.), at boundary of E1/M2 (2nos.), M2 (1no) and at E2 domains (2nos) of Connexin-46. In human, most mutations seem to be associated with zonular pulverulent opacities featuring nuclear or perinuclear regions. However, other variable phenotypes such as anterior capsular with cortical opacities [45], nuclear [53] and total cataracts [45] have also been encountered. Interestingly, the mutation T19M in the present study and the one in an earlier report (T87M) are missense mutations substituting a polar amino acid (Thr) for a non-polar one (Met); they are phenotypically different in a way that might reflect their domain location.

In rats, a non-conservative missense mutation was reported (Glu42Lys [54]), and a knockout mutation of *Gja3*^−/−^ in mouse causes nuclear opacity [55]. *Gja3* knock outs in mice present various degrees of cataractous opacity depending on the genetic background [40] as was also seen with the intra familial variation in the Chinese family. Murine *Gja8* dominant as well as null mutations also display different types of cataracts [56,57]. On the contrary to the mutations mapped in *Gja8,* all missense mutations in *Gja3* are dominant as discussed earlier [13]. Therefore, it is well documented that genetic background does influence the severity of cataract phenotypes with connexin mutations [58]. Moreover, electrophysiological studies of the *Gja8*-G22R mutation at the NH_2_-terminal region have confirmed that mutant subunits alter the gating and conductance of gap junction channels in vitro [59]. Similar experiments on the new T19M GJA3 mutant protein might help to understand the biophysical properties of the mutant connexin-46 molecule in conjunction with the wild type protein.

In conclusion, we present here ample evidence for new cataract-causing mutations in *CRYBB2* and in *GJA3*. The data presented focuses on the complexity of genetic and phenotypic diversity of congenital or childhood cataracts in humans.
